# Predicting Functional Gene Links from Phylogenetic-Statistical Analyses of Whole Genomes

**DOI:** 10.1371/journal.pcbi.0010003

**Published:** 2005-06-24

**Authors:** Daniel Barker, Mark Pagel

**Affiliations:** School of Animal and Microbial Sciences, University of Reading, United Kingdom; Weill Medical College of Cornell University, United States of America

## Abstract

An important element of the developing field of proteomics is to understand protein-protein interactions and other functional links amongst genes. Across-species correlation methods for detecting functional links work on the premise that functionally linked proteins will tend to show a common pattern of presence and absence across a range of genomes. We describe a maximum likelihood statistical model for predicting functional gene linkages. The method detects independent instances of the correlated gain or loss of pairs of proteins on phylogenetic trees, reducing the high rates of false positives observed in conventional across-species methods that do not explicitly incorporate a phylogeny. We show, in a dataset of 10,551 protein pairs, that the phylogenetic method improves by up to 35% on across-species analyses at identifying known functionally linked proteins. The method shows that protein pairs with at least two to three correlated events of gain or loss are almost certainly functionally linked. Contingent evolution, in which one gene's presence or absence depends upon the presence of another, can also be detected phylogenetically, and may identify genes whose functional significance depends upon its interaction with other genes. Incorporating phylogenetic information improves the prediction of functional linkages. The improvement derives from having a lower rate of false positives and from detecting trends that across-species analyses miss. Phylogenetic methods can easily be incorporated into the screening of large-scale bioinformatics datasets to identify sets of protein links and to characterise gene networks.

## Synopsis

A typical fully sequenced genome from a bacterial species contains several thousand genes, and those from multicellular animals may contain many thousands of genes. Understanding the function of these genes is one of the key goals of the developing fields of bioinformatics and proteomics, and the results are of interest to life scientists. The authors describe a computational statistical method that can identify pairs of genes whose functions may be linked, in the sense of participating in a common metabolic pathway or from some physical interaction. The method is applied to phylogenetic trees of related organisms and identifies instances in which a pair of genes is either gained or lost together during evolution. They find that genes that have co-evolved like this on two or more occasions during their evolutionary history are almost certainly functionally linked. These methods can be applied in an automated way to large numbers of species for which fully annotated genomes are available to identify candidate sets of functionally linked genes, and to characterize gene networks.

## Introduction

Evidence that two or more traits co-evolve across a range of species can be used to test hypotheses about the common selective pressures acting on the traits, and about the functional or adaptive relationship between them. Correlated evolution is increasingly being applied at the genetic level on the premise that genes that are gained and lost together [1–3], or that show similar expression patterns or rates of evolution [4,5], may form a functional linkage. This provides a computational approach that can screen large genomic datasets for functional links [6] and help to identify the functions of uncharacterised genes. Such analyses can also be used to describe metabolic networks [7], and discover gene “modules” or clusters of genes engaged in a common function [8].

**Figure 1:**
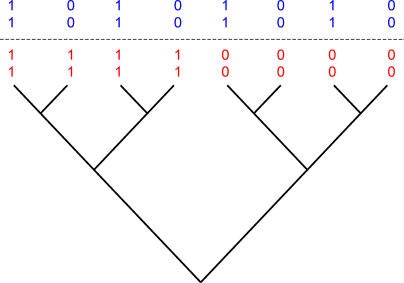
Across-Species Correlation Confuses Shared Inheritance with Correlated Evolution but Phylogenetic Method Does Not The figure shows a hypothetical phylogeny of eight species. Assume all four genes were present in the common ancestor. Only the top (blue) pair provides statistical evidence for correlated evolution. The apparent correlation in the bottom (red) pair arises from shared inheritance of the loss (state “0”) of both genes in the ancestor to the four species on the right of the diagram. Although the two genes were lost at the same time, it may have been for unrelated reasons. By comparison, the correlation in the top pair rests upon four separate events of the correlated loss of both genes. Both genes are retained until near the tips of the tree, at which point both are lost in each of four separate species. It is unlikely that two genes would be simultaneously lost on four independent occasions, unless the two genes were functionally linked. A simple across-species correlation does not discriminate between these two scenarios, whereas one that accounts for phylogeny does. This is an extreme scenario but many others are possible.

Genes and their expression patterns evolve in a phylogenetic context such that functional links of adaptive value tend to be conserved and inherited by descendant species. Among closely related species, shared phylogenetic inheritance can also produce correlated gene profiles for genes that are not linked. Two or more genes might arise independently in a common ancestor and be retained in evolutionary descendants owing to their individual adaptive functions. Figure 1 (numerals in red) shows how this can produce spurious evidence of a functional link when measured across species. By comparison, multiple independent phylogenetic events of the gain/loss of pairs of genes make a compelling statistical case for a functional link (Figure 1, numerals in blue). Phylogenetic methods have uses beyond merely accounting for shared inheritance: they make it possible to investigate ancestral states and to identify the probable temporal ordering of changes in two traits. Knowledge of which of two traits changed first in the evolutionary history the phylogeny describes can be used to test ideas about cause and effect or the dependency of one trait on another [9,10].

Our interest is to evaluate whether incorporating phylogenetic information improves the identification of functional gene links. The need to take account of phylogenetic relationships in comparative studies has long been appreciated in evolutionary biology [11,12] but has received less attention in bioinformatics studies [1,2]. We apply the phylogenetic-statistical method Discrete [9], for assessing correlated evolution among pairs of discrete traits, to data on the presence and absence of pairs of genes. The method identifies independent events of correlated evolution on a phylogeny by comparing the statistical likelihood of the observed data under two alternative scenarios, one in which the two genes are allowed to evolve on the phylogeny independently, and another in which they co-evolve. Trait evolution is modelled as a continuous-time Markov process, and evidence for the model of correlated evolution is assessed by means of the likelihood ratio statistic.

Our dataset consists of a phylogeny of 15 eukaryote species for which complete or nearly complete sequenced genomes are available. There is no limit to the number of species that can be used, but it is important to use fully sequenced and well-annotated genomes to ensure that genes determined to be “absent” are in fact not in the genome. We compare the phylogenetic method's predictions to predictions derived from across-species correlations; the latter have been used in bioinformatics investigations to predict functional gene links [1,2]. We use the Munich Information Center for Protein Sequences (MIPS) [13] database of annotated complexes of yeast proteins as a “known” criterion measure. The MIPS functional links have been determined by low-throughput laboratory procedures and therefore provide a reliable collection of functional links in this species. We find that incorporating phylogenetic information improves predictions by up to 35% over across-species correlations in detecting functional links, and increasingly so for pairs of genes with greater phylogenetic evidence of a functional link. The number of times a pair of genes has been independently gained or lost on the phylogeny is a strong predictor of functional linkage, such that protein pairs with at least two to three correlated events are almost certainly functionally linked.

## Results

### Phylogenetic Tree

**Figure 2:**
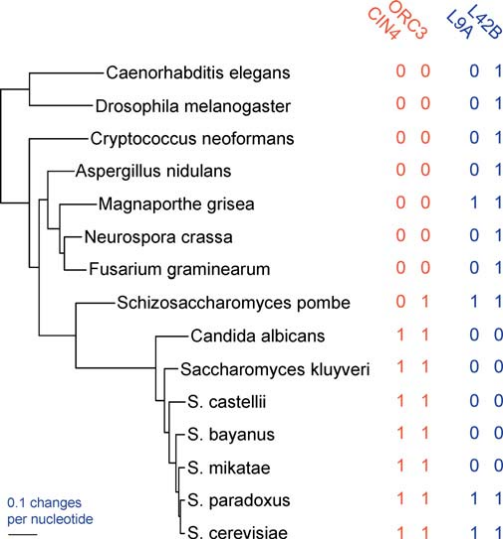
Phylogeny of the 15 Species Showing Two Pairs of Presence/Absence Data for Proteins in MIPS All nodes of the tree received 100% posterior support in an MCMC analysis (see Results). The protein pairs {CIN4, ORC3} and {L9A, L42B}, marked “1” for presence and “0” for absence, are included to illustrate probable type I (false positive) and type II (false negative) errors by the across-species method in real data (see Results, “False positives”). Probable false positive: The across-species correlation returns a significant (*p* = 0.0014) correlation between the pair {CIN4, ORC3}. The phylogenetic method regards this as a chance association (*p* = 0.13) arising from a single event of both genes being gained in the ascomycete yeasts, followed by shared inheritance (as in Figure 1). The pair {L9A, L42B} consists of two functionally linked proteins. These return a significant phylogenetic correlation (*p* = 0.035) owing to perhaps five correlated losses of both genes (see text). The across-species association is sensitive only to the distribution of the two proteins across the tips, and returns a non-significant result (*p* = 0.23). This is a probable false negative.

Figure 2 shows the maximum likelihood phylogenetic tree of the 15 species. We also conducted Bayesian Markov chain Monte Carlo [14] phylogenetic analyses [15–21] using the program BayesPhylogenies [21]. The posterior support as derived from the Markov chain Monte Carlo (MCMC) analysis was 100% at all nodes, and our topology for the yeast species agrees with a recent yeast phylogeny [22]. This is not to say that either our tree or that in [22] is the “true” tree (compare to [23]), just that given our data and model of evolution, no other tree was sufficiently likely to be included in the posterior Bayesian sample.

### Distribution of Likelihood Ratios

**Figure 3:**
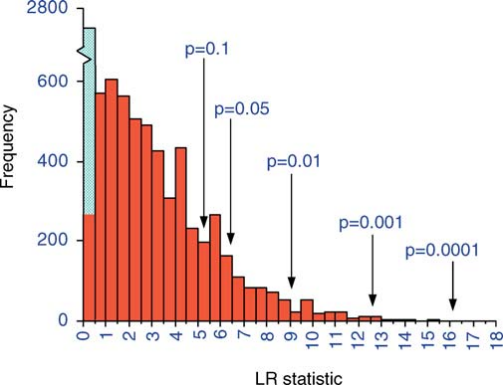
Distribution of 8,102 LRs for MIPS Pairs, Measuring the Strength of Support for the Phylogenetic Correlation Critical *p*-value cut-off points are derived from the random pairs data (see Results). The blue bar within the first class represents the 2,483 pairs for which one or both proteins were present in all 15 species (LR ≈ 0). The red bars record the remaining 5,619 LRs for pairs of proteins that both vary across species. Approximately 8% of the results exceed the *p* ≤ 0.05 level. Two pairs have LRs greater than 15.5 but are not visible on the graph. The excess of LR scores of 4–4.5 and 5.5–6 may arise from misidentified homology in *S. kluyveri*. This species is identified as having a smaller number of genes than its phylogenetic neighbours. These paired absences will tend to inflate correlations. We left these results in our analyses, as they affect the phylogenetic and across-species analyses equally, and we cannot be sure which absences are real and which are not.

We calculated the likelihood ratio statistic (see Materials and Methods) to test for correlated evolution in 10,551 pairs. We excluded pairs that yielded any evidence of a negative relationship (*n* = 2,449) on grounds that one gene being present when the other is absent cannot be evidence for a functional link. Figure 3 shows the distribution of the remaining 8,102 likelihood ratios. Larger values of the likelihood ratio (LR) statistic provide stronger evidence for correlated evolution. The blue bar identifies 2,483 likelihood ratios corresponding to pairs of genes in which one or both genes are present in all 15 species and therefore cannot be studied for evidence of correlated evolution. The red bars exclude these pairs, leaving 5,619 LRs for which both genes vary across species. This skewed distribution has a mean of 3.36 ± 2.47, with two values greater than 15.5.

To assign *p*-value cut-off points to the distribution of varying protein pairs, we simulated the null LR distribution from 9,509 random protein pairs from the MIPS database. These pairs were drawn to have the same across-species distribution as the MIPS pairs, but with the restriction that none of the random pairs came from the same protein complex. The 9,509 pairs produced 6,393 likelihood ratios for pairs of genes that both vary across species, of which 3,722 represent a non-negative relationship. This set of likelihood ratios is well characterized by a gamma probability density distributed as *GAM*(1.9,1.4). The *p*-value cut-off points shown on Figure 3 are derived from this null-distribution. Although our expectation is that the random pairs do not represent true links, they are likely to be a conservative control on the MIPS pairs, as some may describe as-yet undiscovered functional links.

Of the MIPS pairs whose patterns both vary across species, 609 (11%) have LRs that exceed the *p* ≤ 0.05 level. Among these, 185 (3.3% of the total) exceed the *p* ≤ 0.01 level. There were *n* = 278 pairs for which both genes are found in all 15 species. If these are assumed to represent functional links, then the total number of across-species functional links remains around 11% = (609 + 278)/8,102; we use 8,102 in the denominator, because we are now including the constant pairs in the calculation. Varying pairs contribute roughly twice as much to this total as do the constant pairs. Even in this hand-picked dataset of known interactions in *Saccharomyces cerevisiae,* only a comparatively small number generalise across species.

### Detection of “Known” Protein Interactions

We wish to know whether the LR method gets better at detecting “known” interactions the more extreme its statistical result. We combined the 8,102 LRs corresponding to all non-negative MIPS relationships, with the 6,838 LRs obtained from the randomly generated pairs in which the relationship is also non-negative. We then assigned the combined data to *p*-value bins and determined the percentage of the results in each bin that correspond to MIPS pairs. If large values of the likelihood ratio are indicative of functional links, then this percentage should increase as the *p*-value declines. If, however, random pairs are just as likely to show large *p*-values, the percentage will not improve. To measure the influence of adopting a phylogenetic perspective, we compared the phylogenetic LR method's performance at identifying true MIPS pairs with the across-species correlation (Fisher exact test, but again excluding pairs with a negative correlation).

**Figure 4:**
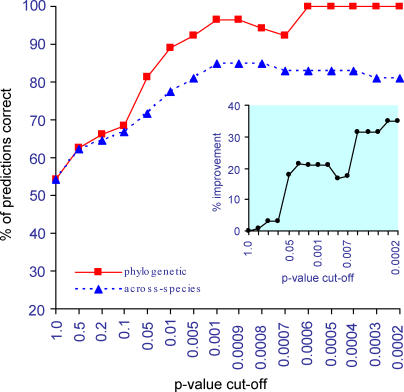
Phylogenetic Method Identifies a Higher Percentage of Functional Links than the Across-Species Correlation The main graph shows the percentage of the predicted links at or below a given p-value, that correspond to annotated functionally linked pairs in the MIPS database, separately for the two methods. At a *p*-value of 1.0 or less, both methods declare all of the pairs to be functionally linked, producing a correct percentage of 54% (see Results). Inset: the percentage by which the phylogenetic method improves upon the across-species correlation, where improvement = (percent correct phylogenetic − percent correct across-species)/54.

Figure 4 compares the two methods' performance, plotting the percentage of the predicted links at or below a given *p*-value that correspond to annotated functionally linked pairs in the MIPS database. At *p* ≤ 1.0, both methods declare every pair significant producing a correct rate of 8,102/(8,102 + 6,838) = 54%. As the *p*-value decreases, the percentage of results that are MIPS pairs increases for both methods. The phylogenetic method correctly classifies a higher percentage of the pairs than the across-species correlation at each *p*-value level, and eventually includes only MIPS pairs in its predictions. By comparison, the across-species correlation method reaches a plateau beneath 100% correct.

The inset graph in Figure 4 plots the phylogenetic method's relative improvement over the across-species correlation. The phylogenetic LR shows a pronounced rise at the *p* ≤ 0.05 level, to 18% improvement, increasing to 35% improvement at more extreme *p*-values. This is the direct contribution of taking into account shared phylogenetic inheritance. At a *p*-value of about 0.0006 or less, all of the pairs that the phylogenetic LR method identifies represent known functional links. LRs significant at this level roughly correspond to at least two to three phylogenetically independent pairs of gain/loss events on our phylogenetic tree. This suggests that a consistent pattern of phylogenetic co-evolution almost certainly points to a functional link, and increasingly so as the number of co-evolutionary events increases. The across-species test has no way to discriminate multiple independent events from a single event that is retained and inherited by many species (see Figure 1). This causes it to misclassify many pairs and to fail to improve at identifying functional links even at more extreme *p*-values.

### False Positives

**Figure 5:**
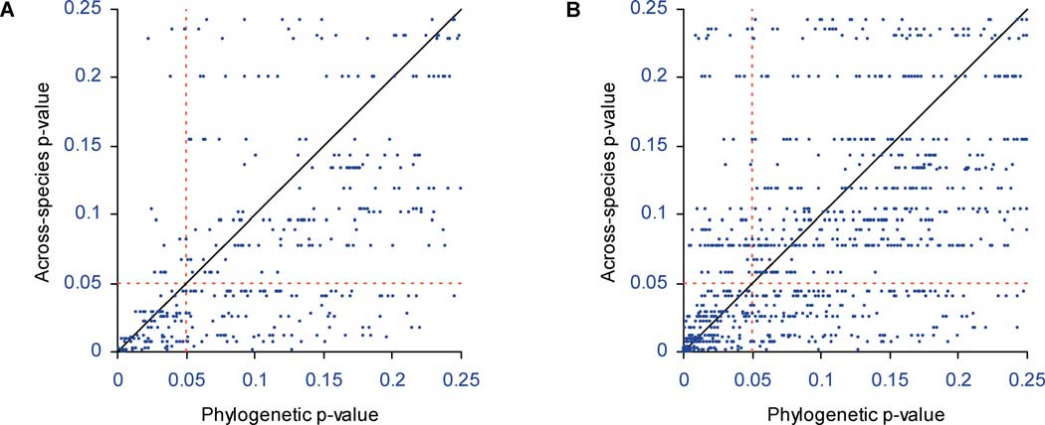
Phylogenetic Method Results in Fewer False-Positives than the Across-Species Correlation The across-species *p*-value (y-axis) is plotted against the phylogenetic method's *p*-value (x-axis) for the range of *p* = 0–0.25; the methods draw similar conclusions for *p*-values greater than 0.25. (A) Higher rates of probable false positives for the across-species correlation. The horizontal dashed line defines the region in which the across-species method declares pairs significant (*n* = 170) but the phylogenetic method finds no evidence for a functional link. The vertical dashed line defines the same region for the phylogenetic method (*n* = 32). (B) Same relationship as in (A) but for the MIPS pairs of annotated links. The across-species correlation returns a functional link for *n* = 278 pairs that the phylogenetic method declares non-significant. Many of these may be false positives arising from chance events (see Results). The phylogenetic method finds *n* = 186 extra pairs significant. Especially at lower *p*-values, these are unlikely to be false positives (see Results) (Figure 4).

If false positives cause the across-species correlations to classify a lower percentage of the true pairs correctly, this should be apparent from comparing the two methods in the random-pairs data. Figure 5A plots the across-species *p*-value against the phylogenetic LR *p*-value for pairs of proteins in the random-pairs dataset. The correlation between the two methods' *p*-values is *r* = 0.85 for all pairs. Removing the 3,116 pairs in which at least one gene is found in all 15 species and thus both methods return a *p*-value of 1.0, the correlation falls to *r* = 0.73. This means that the methods have only 53% of their variance in common and shows that they respond to different aspects of the data. The diagonal 1:1 line indicates that the across-species *p*-values tend to fall at or below (more extreme than) the LR *p*-value; it declares more of the pairs to be functionally linked. The horizontal line through the *p* = 0.05 level identifies 170 protein pairs in this random pairs data that the across-species correlations declare significant, but the phylogenetic LR method finds not significant. Many of the LRs in this region have large *p*-values, indicating that there is no evidence of repeated independent events of correlated change. By comparison, the vertical line through *p* = 0.05 shows that in only 32 cases does the LR method declare a result significant that does not show a significant across-species pattern. Taken together, these results illustrate how the across-species correlation is prone to picking false positives.

Figure 5B plots the same comparison, but this time for the MIPS pairs. The across-species correlation again tends to have lower *p*-values but the trend is less pronounced. The correlation between the two methods is *r* = 0.86 or 74% shared variance for all patterns, and *r* = 0.74 or 55% shared variance for pairs in which both genes vary across species. The two methods agree on 423 pairs at the *p* ≤ 0.05 level. The horizontal dashed line identifies 278 pairs that the across-species correlation declares significant but the LR method finds not significant. Are these false positives? The results in Figure 5A show that the across-species correlation is often misled by shared phylogenetic inheritance. It is tempting to speculate, therefore, that many of these 278 results are false positives, even though they are linked in *S. cerevisiae*. The vertical dashed line shows that the phylogenetic LR method identifies 186 pairs as significant that the correlation method declares not significant. Figure 4 suggests that LR method's extra 186 MIPS pairs are unlikely to be false positives.

The LR method's improvement over the across-species correlation seems principally to derive from correctly excluding spurious functional links that arise from shared phylogenetic inheritance, but also from correctly identifying some patterns of co-evolution that the across-species correlation misses. The two pairs of proteins shown alongside the phylogeny in Figure 2 illustrate this point. The across-species correlation is significant (*p* = 0.0014) between the pair {CIN4, ORC3}, whereas the phylogenetic method regards this as a chance association (*p* = 0.13) arising from a single event of both genes being gained in the ascomycete yeasts, followed by shared inheritance (like the red distribution patterns in Figure 1). In agreement with the phylogenetic approach, the proteins' known functions do not suggest a link. In contrast, the pair {L9A, L42B} consists of two proteins that are functionally linked, as components of the cytoplasmic ribosomal large subunit. The pair returns a significant phylogenetic correlation (*p* = 0.035). The across-species correlation is sensitive only to the distribution of the two proteins across the tips of the tree and returns a non-significant result (*p* = 0.23). If we assume that independent gains of the same gene are unlikely, then L9A was present relatively early on in the phylogeny, no later than the common ancestor to the *Aspergillus nidulans-S. cerevisiae* clade. It was lost in *A. nidulans* and separately in the *Neurospora crassa-Fusarium graminearum* group, even though L42B was present. L42B and L9A were lost together on five separate occasions spanning *Candida albicans* to *Saccharomyces mikatae,* but both were retained in *S. paradoxus* and *S. cerevisiae*. The across-species correlation is not sensitive to these changes, and its result is probably a false negative or type II error.

### Contingent Gain or Loss of Genes

Contingent relationships between a pair of genes describe cases in which one gene is more likely to be gained or lost depending upon the state of the other. One example of this might be cases in which two genes are paralogues, and so one of the pair gets lost in each species owing to its redundant function. Other cases might identify instances in which one gene's function depends upon the presence of a second gene, but the second gene performs functions even in the absence of the first. Such contingent linkages may describe and explain many of the large number of cases in which two genes are functionally linked in one species, but they do not exclusively appear together across species. They can be detected by estimating the transition rate parameters of the dependent model (see Materials and Methods) [9] and looking for rates of evolution of one gene being dependent upon the presence or absence of the other.

**Figure 6:**
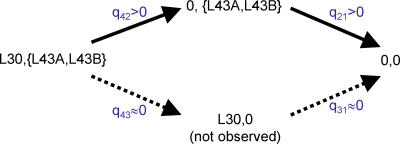
Detecting Contingent Evolution Between Two Proteins Protein L30 is significantly linked across species to L43A and L43B (both LRs = 9.73, *p* < 0.007). The three are present together in nine of the species, and are probably ancestral to the group represented by the phylogeny in Figure 2. The diagram represents the probable ancestral states on the left side. Solid arrows indicate the most likely events of evolution to other evolutionary states, and dashed arrows correspond to events for which no statistical support is found. L30 can be lost (*q_42_* > 0), leaving L43 and L43B remaining, and this happens in two species. Once L30 is lost, the other two proteins follow (*q_21_* > 0), yielding the remaining four species in which both proteins are absent. In comparison, L43A and L43B are never lost in the presence of L30. This suggests a contingent relationship amongst these proteins such that L43A and L43B seem to derive their functions only in the presence of L30.

Three cytoplasmic ribosomal large subunit proteins may provide an example of contingent evolution. Protein L30 is significantly linked to proteins L43A and L43B: both LRs = 9.73, *p* < 0.007. L43A and L43B are duplicates with identical protein sequence, and L30 is auto-regulatory [24]. The three proteins are present together in nine of the species, and are probably ancestral to the group represented by the phylogeny in Figure 2. Figure 6 represents this scenario on the left side of the diagram as all three proteins present. The remainder of the figure shows a model describing the contingent manner in which these ancestral proteins are lost. Solid arrows indicate most likely events of evolution to other evolutionary states, dashed arrows correspond to events for which no statistical support is found. See Materials and Methods for details of the transition rates *q_ij_*. L30 is lost (*q_42_* > 0) in two species while leaving L43A and L43B remaining. Once L30 is lost, the other two proteins follow (*q_21_* > 0), yielding four species in which both proteins are absent. In comparison, L43A and L43B are never lost when L30 is present (*q_43_* is not significantly different from zero). This may indicate a dependent relationship amongst these genes such that L43A and L43B acquire their function in the presence of L30.

## Discussion

Incorporating phylogenetic information into predictions of functional gene links improved by between 18% and 35% upon predictions derived from across-species correlations, and increasingly so for pairs of genes with greater evidence of correlated evolution on the phylogeny. The phylogeny makes it possible to discriminate across-species patterns that arise by chance through common ancestry from those that indicate multiple independent instances of the correlated gain or loss of a pair of genes. This has implications for methods such as “phylogenetic profiling” [1,2], which, despite its name, does not make use of phylogenetic information when deriving predictions about functional links. In addition to reducing the number of false positives, incorporating phylogenetic information can sometimes recognize a true functional link even when the simple across-species pattern is vague and non-significant.

We find that the pairs of genes that have been gained or lost together on two to three or more occasions are almost certainly functionally linked. To our knowledge, this is the first phylogenetic demonstration that correlated evolutionary events strongly imply functional linkage, and underscores the importance of analysing events of protein evolution on phylogenetic trees. As the number of fully sequenced genomes increases, phylogenetic approaches can be used with increasing sensitivity to detect multiple events of correlated gene evolution, and by inference, pairs of genes with a high probability of being functionally linked.

We studied functional links on only a single phylogenetic tree rather than on a sample of trees, because we wished to compare results to the across-species correlation, which has no way of making use of the phylogenies. But it is straightforward to implement our approach in a Bayesian framework such that functional links are estimated across a sample of trees. Elsewhere we describe how to derive Bayesian posterior probability distributions of the parameters of the continuous-time Markov model of trait evolution, estimated over the posterior probability distribution of phylogenetic trees [25,26]. This accounts for uncertainty about the tree and about the parameters of the model of trait evolution, and can be especially valuable where there are disagreements about the placement of some species or groups of species.

A surprising number of gene pairs that are annotated as functionally linked in yeast do not appear to be linked in other, often closely related, species. Some of these may arise because a gene characterised as “absent” has simply gone unnoticed. We think this is only a small part of the explanation here, as we restricted ourselves to well-annotated, fully sequenced genomes. More likely is that the set of across-species functional links is far smaller than the set of all known links within any given species, and this raises the question of just what an across-species functional link measures. One distinct possibility is that a fundamental set or “backbone” of conserved protein interactions exists, in what might be called the “correlated evolution network.” This set of links is distinctive, in that the pairs of genes tend either to be both present or both absent. If so, their identification should be given a high priority, as they may reveal general organismic “rules of assembly.”

The highly specific nature of functional links also has implications for using model organisms to make predictions about other species, such as humans. Our data suggest that such predictions will often be wrong: Many genes whose functions and links have been identified from in-depth study in a model species may adopt different functions in other species. A phylogenetic method routinely applied to large numbers of species could distinguish the subset of genes whose functions can be reliably assumed to generalise from those that do not. Used in combination with low-throughput single-species studies, a more sophisticated picture may emerge.

In any analyses relying on identification of orthologues across species, multigene families may cause particular headaches. Assuming that the functionally conserved orthologue of a given gene will be under similar selection pressures and therefore have the greatest sequence similarity on average, reciprocal sequence similarity procedures such as we have used (see Materials and Methods) should perform well. Because the possibility of mis-identification can seldom be ruled out with certainty, additional evidence for correct annotation should be sought when a gene is suspected to be part of a larger family. Another approach is more practical: Simply exclude genes from consideration if they appear in multiple copies in a target species [27].

A large number of genes remain uncharacterised. Identifying functional linkages from phylogenetic events of co-evolution with other genes seems a promising way to understand function, and is an approach that can yield insights from currently poorly understood genomes. It is encouraging that we are able to detect functional links with reasonable sensitivity and specificity in a comparatively small number of species. Larger datasets will not only improve the ability to detect correlations; they will also make it possible to link events of correlated evolution to background organismic and ecological variables, and to identify clusters of genes that tend to appear together. Our approach can also be easily modified to use continuously varying data. Such data are increasingly becoming available from sequence similarity searches [3] and micro-array expression studies, and may provide a rich source of information on functional linkages and the nature of mRNA expression evolution [28].

## Materials and Methods

### 

The method requires a phylogeny of the organisms to be investigated, plus data on the presence and absence of homologous genes.

#### Gene-sequence data for phylogenetic tree.

All of our analyses are conducted on a phylogenetic tree of fifteen eukaryote species for which whole-genome data were available in 2003, including the 13 fungal species *S. cerevisiae, S. bayanus,* *S. mikatae,* *S. paradoxus, S. castellii,* and* S. kluyveri* (available from the *Saccharomyces* Genome Database, at ftp://ftp.yeastgenome.org/yeast/);* C. albicans* (Stanford Genome Technology Center, at http://www-sequence.stanford.edu/group/candida); *S. pombe* (Wellcome Trust Sanger Institute, at ftp://ftp.sanger.ac.uk);* A. nidulans, F. graminearum,* and* M. grisea* (Broad Institute, at http://www.broad.mit.edu); *N. crassa* (MIPS, at ftp://ftpmips.gsf.de; also the Broad Institute); and *Cryptococcus neoformans* (preliminary sequence data obtained through The Institute for Genomic Research, at http://www.tigr.org). In addition, genome data for two animals were used: *C. elegans* (Wormbase, at http://www.wormbase.org) and* D. melanogaster* (Berkeley Drosophila Genome Project, at http://www.fruitfly.org). Where two predicted protein sets existed for the same species (i.e., *S. bayanus*, *S. mikatae,* and *N. crassa*), these were combined into a single nonredundant set.

We used gene sequences for EF-1 alpha and EF-2 to infer the phylogenetic tree. We obtained proteins and their corresponding nucleotide sequences for each species, and aligned the data at the protein level using Clustal-X [29] before converting it back to nucleotides with Protal2DNA (http://bioweb.pasteur.fr). Ambiguously aligned codons were detected by eye and removed, yielding 1,425 aligned nucleotide sites.

#### Phylogenetic inference.

We reconstructed the phylogeny using a general time-reversible model of sequence evolution and allowing for gamma-distributed rate-heterogeneity (GTR+Γ). We found a tree for the 15 species using a heuristic search for the maximum likelihood (ML) tree in PAUP, and we refer to this as the ML tree. For comparison, we sampled the posterior probability distribution of phylogenies using the same model of evolution in a Bayesian MCMC framework (as described in [21] and using the program BayesPhylogenies). Bayesian methods are becoming increasingly popular in phylogenetic studies, providing a statistically rigorous way to describe uncertainty about the true phylogeny. After discarding the first 300,000 trees in the chain as a “burn-in” period, we sampled 500 trees at intervals of 50,000 trees to ensure that successive trees in our sample were independent (autocorrelation of log-likelihoods = 0.00). There was no increase in the mean log-likelihood after burn-in. The consensus tree topology from the posterior distribution was identical to the ML tree, with 100% posterior support at all nodes.

We used the single ML tree in all of our analyses of correlated evolution, rather than calculating correlated evolution across the Bayesian sample [25]. The latter approach is preferable in that it accounts for any effects of phylogenetic uncertainty on our results. In the present case, that uncertainty is limited to variation in branch lengths, because only one tree topology was found in the Bayesian sample. Variation in branch-lengths can influence likelihoods, but our interest is to compare the performance of phylogenetic and across-species methods, and the across-species method has no way of using the extra information in the Bayesian sample of trees.

#### Gene presence/absence data.

The MIPS database lists 260 known *S. cerevisiae* protein complexes and the 1,156 proteins that form them. Regarding each protein within a complex as functionally linked to every other different protein in that complex gives 10,551 pairwise links. For each of these *S.* *cerevisiae* proteins, we sought orthologues in the 14 other species. We took a reciprocal best-in-genome global alignment between proteins to indicate “presence” of an orthologue and no such reciprocal hit to indicate “absence.” A heuristic approach was used (conceptually based on [31]), in which up to 20 best local alignments were obtained with BLASTP [32], then scores were re-assigned using Needleman-Wunsch alignment (EMBOSS Needle [33]).

#### Statistical modelling of correlated gene evolution.

We modelled correlated gene presence/absence on a phylogeny using a continuous-time Markov model [9,34]. The method compares the statistical likelihood of a model in which two binary traits are allowed to evolve independently on the tree, with a model in which the two traits are allowed to evolve in a correlated fashion. Evidence for correlated evolution arises if the dependent or correlated model shows a significantly better fit to the data than the independent model. The method has been applied to molecular evolution studies of prion proteins [35] and co-evolving protein residues [36]; and to estimating ancestral states of artiodactyl ribonucleases [25,37], rates of change in homing endonucleases [38], and the history of lichenization in fungal evolution [20].

We describe the method in some detail below, as our presentation only partially overlaps with that in [9], and elements of our description are pertinent to modelling gene presence/absence data. Here the binary trait is the presence or absence of each gene as observed in the species at the tips of the phylogeny (see, for example, Figure 1). Two binary traits can produce four different pairs of outcomes or states, corresponding to the pairings of presence or absence in two genes. The diagram in Equation 1 links the four states by arrows with parameters that describe the rates of transition between the two states of one of the genes, holding the state of the other constant. If two traits evolve independently of one another, then the rate of change between the presence (“1”) or absence (“0”) of one gene will not depend upon whether the other is present or absent. For example, if the rate of change from state “0” to state “1” in the second gene does not depend upon the state of the first variable, then *q_12_* will be equal to *q_34_*. More generally, the model of independent evolution implies that *q_13_* *=* *q_24_,* *q_42_* =* q_31_,* *q_43_* *=* *q_21_,* and *q_12_* = *q_34_* and therefore requires a maximum of four parameters.


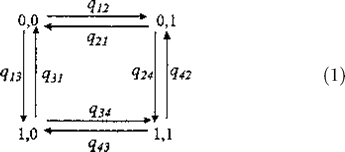


The model of correlated evolution does not place any restrictions on the parameters, using a maximum of eight parameters to describe the data. The correlated evolution model will improve on the independent model when the distribution of the traits across the species of the phylogeny implies that some of the pairs of transition rates constrained in the independent model to be equal to each other, in fact differ. Information that pairs of coefficients differ arises not from the number of species that come to inherit a particular set of outcomes, but from the implied number of times the events represented by the rate coefficients have occurred on the tree. This is how the likelihood approach discriminates between the two scenarios of Figure 1.

The method is formally described by a rate matrix **Q:**


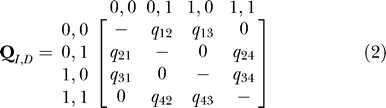


where we use the **Q** *_I,D_* notation to indicate that the matrix can be configured to either the independent or dependent (correlated) model depending upon whether some pairs of transition rates are constrained to be equivalent. The main diagonal elements are defined as minus the sum of the other rate coefficients in the row of the matrix, such that each row sums to zero. The values of all dual transitions, or cases in which both traits change simultaneously, are set to zero in the matrix in Equation 2. Dual transitions are set to zero because their transition rate parameters measure the probability of both traits changing simultaneously in infinitesimally short interval *dt*. The probability of both traits changing in the same instant is negligibly small and can be ignored.

The model does allow both traits to change over a longer interval *t,* however. Thus if the four states in the matrix above are numbered 0, 1, 2, and 3, we can write the probability of a change in the short interval *dt* as *P_ij_(dt)* = *q_ij_dt,* where *i* and *j* can take the values 0 to 3. The probabilities over longer intervals *t* are found by exponentiating the **Q** matrix multiplied by the length of the interval:


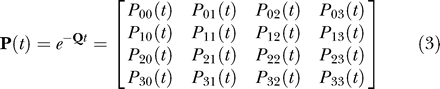


Combining these probabilities over all branches of the tree yields the likelihood of the data, *L*. The method of ML finds the values of the rate parameters in **Q** that make the observed data most probable, given the **Q** and the phylogenetic tree.

The likelihood is summed over all possible ancestral state reconstructions [9,39], meaning that results do not depend upon any particular inferred evolutionary history of the traits. In the case of gene presence/absence data, this can mean that a gene is allowed to arise or evolve more than once on the tree, something that is probably highly unlikely except in the case of lateral gene transfer. In practice, we expect that most correlated evolution takes the form of coincident losses of genes. However, given that homology is assessed as similarity at the sequence level, small amounts of convergent evolution could make two initially dissimilar sequences become more similar and thereby appear as orthologues. To exclude or reduce the effect of allowing multiple gains among highly diverged species, we fixed the root of the tree at “present” for any gene that was found on both sides of the major bifurcation that the root defines. This causes the model to favour losses for those pairs.

The Discrete method can be used with a single tree and is now implemented in a Bayesian framework in the program BayesDiscrete, to account for uncertainty in both the estimates of the phylogeny and in the parameters of the model of correlated evolution. It is available from M. Pagel and A. Meade at http://www.ams.reading.ac.uk/zoology/pagel.

#### Hypothesis testing.

When the independent and dependent models are estimated by maximum likelihood, their goodness of fit is compared using the LR statistic: LR = −2 log*(H_0_)* − log*(H_1_)*, where *H_0_* is here the likelihood of the model of independent evolution and *H_1_* is the model of dependent evolution. This test statistic is asymptotically distributed as a χ^2^ variate with degrees of freedom equal to the difference in the number of parameters of the two models (here four). If LR exceeds the critical value of the χ^2^ distribution with appropriate degrees of freedom, then the *H_1_* model is judged a better fit to the data.

When the phylogeny contains a small number of species or rates of evolution are low, the LR statistic as defined above is often distributed with fewer than four degrees of freedom [34]. In these cases, the correct null hypothesis distribution can be simulated following [9,34].

#### Across-species correlation.

We used Fisher's exact test (e.g., [40]) to analyse the 2 × 2 table, recording the number of species with each of the four possible categories of presence/absence of two genes. This test makes no distributional assumptions and returns an exact *p*-value for the null hypothesis that the two traits are distributed independently.

## Supporting Information

### Accession Numbers

Swiss-Prot (http://www.ebi.ac.uk/swissprot/) accession numbers for the yeast proteins discussed in this paper are CIN4 (P39110), L9A (P05738), L30 (P14120), L42B (P02405), L43A (P49631), L43B (P49631), and ORC3 (P54790).

## Abbreviations

LR: likelihood ratio
MCMC: Markov chain Monte Carlo
MIPS: Munich Information Center for Protein Sequences
ML: maximum likelihood
